# Primary tumour location affects survival after resection of colorectal liver metastases: A two-institutional cohort study with international validation, systematic meta-analysis and a clinical risk score

**DOI:** 10.1371/journal.pone.0217411

**Published:** 2019-05-31

**Authors:** Elisabeth Gasser, Eva Braunwarth, Marina Riedmann, Benno Cardini, Nikolaus Fadinger, Jaroslav Presl, Eckhard Klieser, Philipp Ellmerer, Aurélien Dupré, Katsunori Imai, Hassan Malik, Hideo Baba, Hanno Ulmer, Stefan Schneeberger, Dietmar Öfner, Adam Dinnewitzer, Stefan Stättner, Florian Primavesi

**Affiliations:** 1 Department of Visceral, Transplant and Thoracic Surgery, Medical University of Innsbruck, Innsbruck, Austria; 2 Department of Medical Statistics, Informatics and Health Economics, Medical University of Innsbruck, Innsbruck, Austria; 3 Department of Surgery, Paracelsus Medical University, Salzburg, Austria; 4 Institute of Pathology, Paracelsus Medical University, Salzburg, Austria; 5 Department of Neurology, Medical University of Innsbruck, Innsbruck, Austria; 6 Liverpool Hepatobiliary Centre, Aintree University Hospital, Liverpool, United Kingdom; 7 Department of Surgical Oncology, Centre Léon Bérard, Lyon, France; 8 Department of Gastroenterological Surgery, Kumamoto University, Kumamoto, Japan; University of Nebraska Medical Center, UNITED STATES

## Abstract

**Background:**

Colorectal cancer (CRC) represents a major cause for cancer death and every third patient develops liver metastases (CRLM). Several factors including number and size of metastases and primary tumour lymph-node status have been linked to survival. The primary tumour location along the colo-rectum continuum (sidedness) was analysed in first-line chemotherapy trials, where right-sided CRCs showed decreased survival. This association has not yet been clearly established in patients undergoing resection for CRLM.

**Methods:**

Clinicopathological differences in CRLM resections according to sidedness in two Austrian centres (2003–2016) are described and survival is compared through Kaplan-Meier and multivariable analysis. A risk-score is presented with time-dependent receiver operating curve analysis and international validation in two major hepatobiliary centres. Furthermore, a systematic meta-analysis of studies on primary tumour location and survival after CRLM resection was performed.

**Results:**

259 patients underwent hepatectomy. Right-sided CRC patients (n = 59) more often had positive primary tumour lymph-nodes (76.3%/61.3%;p = 0.043) and RAS-mutations (60%/34.9%;p = 0.036). The median overall and disease-free survival was 33.5 and 9.1 months in right-sided versus 55.5 (p = 0.051) and 12.1 months (p = 0.078) in left-sided patients. In multivariable analysis nodal-status (HR 1.52), right-sidedness (HR 1.53), extrahepatic disease (HR 1.71) and bilobar hepatic involvement (HR 1.41) were significantly associated with overall survival. Sidedness was not independently associated with disease-free survival (HR 1.33; p = 0.099). A clinical risk score including right-sidedness, nodal-positivity and extrahepatic involvement significantly predicted overall (p = 0.005) and disease-free survival (p = 0.027), which was confirmed by international validation in 527 patients (p = 0.001 and p = 0.011). Meta-analysis including 10 studies (n = 4312) showed a significant association of right-sidedness with overall survival after resection (HR 1.55;p<0.001). There was no significant association with disease-free survival (HR 1.22;p = 0.077), except when rectal-cancers were excluded (HR 1.39;p = 0.006).

**Conclusions:**

Patients with liver metastases from right-sided CRC experience worse survival after hepatic resection. Sidedness is a simple yet effective factor to predict outcome.

## Introduction

Although mortality of colorectal cancer (CRC) is declining within the last years, it still represents the second most common cause of cancer death in Europe [1] (WHO). About one third of patients develop liver metastases (colorectal liver metastases, CRLM) [2], but only 10–30% [3, 4] are usually eligible to undergo liver resection due to the extent of the disease. Progressive liver surgery eventually combined with interventional oncology techniques enables increased resectability in specialized centres [5–7], but preoperative risk stratification is essential to limit postoperative complications and achieve adequate long-term survival benefit [8–10]. Even though radical liver resection is a potentially curative treatment, more than 50% of these patients develop intrahepatic recurrence [11, 12] and recently reported five-year overall survival (OS) rates after liver resection are in the range of 25 to 58% [12–16]. Established risk scores such as the”Fong” score from Memorial Sloan Kettering Cancer Centre (MSKCC) or the Basingstoke predictive index are routinely applied by clinicians to select patients suitable for hepatic resection in terms of risk-benefit ratio. [17, 18] These risk scores mainly utilize clinical and pathological factors such as number and size of metastases or tumour markers to predict OS and disease-free survival (DFS) after liver resection. Recently, markers of tumour biology and genetics such as the RAS or BRAF mutational status are more commonly included in clinical risk scores and algorithms for oncosurgical treatment [19–22]. In this new genomic era of cancer treatment, the relevance of primary tumour location (“sidedness”)–a term commonly used in former times of colorectal surgery—emerged with a new livery. The large bowel develops from different embryonic origins and molecular features change along the length of the colon-rectum. Advancements in molecular biology knowledge and insights of embryogenesis lead to revive previous research and to clinically divide CRCs in right- and left-sided tumours, representing two separate distinct entities [23, 24]. Right sided colorectal cancer (RCRC) is commonly defined as a tumour located between the ileocecal junction and the transverse colon and left colorectal cancer (LCRC) includes all tumours located from the splenic flexure to the rectum. RCRCs are more often diploid and hypermutated, frequently present with microsatellite instability (MSI), and more often have deleterious mutations of RAS, BRAF and PI3KCa and a serrated signature [25–28]. LCRCs more often develop from the classical adenoma-carcinoma sequence of carcinogenesis with aneuploidy and chromosomal instability, leading to amplification of regions hosting receptor tyrosine kinases such as epidermal growth factor receptor (EGFR) [29–33].

Several studies analysed the predictive effect of primary tumour sidedness in metastatic CRC (mCRC) patients treated with palliative first line chemotherapy with or without targeted therapy. A pooled analysis by Arnold et al. showed that patients with LCRC obtained beneficial OS (HR 0.75, p<0.001) and progression-free survival (PFS; HR 0.78, p<0.001) when anti-EGFR was added to chemotherapy, whereas there was no such effect found in patients with RCRC (OS: HR 1.12, p = 0.38; PFS: HR 1.12, p = 0.36) [34]. In a further meta-analysis of first-line clinical trials by Holch et al., a HR of 1.5 for RCRC regarding OS and 1.3 regarding PFS was observed, clearly indicating an independent influence of sidedness on survival in palliative mCRC patients [35]. However, due to low subsequent curative-intent CRLM resection rates of 15% or less in many first line chemotherapy studies, the relevance of sidedness after resection for CRLM is not directly transferable from previous oncological studies and is still debated in the surgical community with conflicting results [14, 36]. The present study investigates sidedness as a clinical prognostic factor regarding survival after resection for CRLM. First, a two-institutional, retrospective cohort analysis of risk factors for survival was performed. In an aim to establish the applicability of primary tumour location in clinical surgical routine, sidedness was also incorporated in a newly proposed clinical risk score for OS and DFS after CRLM resection. This score was validated with data from two large hepatobiliary centres in Europe and Asia. Finally, the survival results of our own cohort were included in a systematic meta-analysis of published studies.

## Materials and methods

### Retrospective two-institutional analysis

#### Study population and design

This study retrospectively reviewed data from prospectively maintained, auditable databases of two Austrian tertiary referral centres (Medical University of Innsbruck and Paracelsus Medical University Salzburg). All patients undergoing curative intent hepatic resection (R0/R1) for newly diagnosed synchronous or metachronous (>6 months after primary tumour diagnosis) CRLM between 2003 and 2016 were included. Patients with simultaneous, curative-intent colorectal primary tumour surgery (one-stage primary and metastasis) were also included. Patients with palliative / debulking surgery were not recorded, as were cases with previous metastasis surgery for CRLM. Extrahepatic disease (EHD) was no exclusion criteria, when it was included in the curative concept, i.e. pulmonary resection, distant lymphadenectomy or cytoreductive surgery (with/without HIPEC).

The following parameters were extracted from the database and completed through patient record charts or external reports in case of missing data: Age at date of liver resection, sex, American Society of Anesthesiologists (ASA) status, TNM (tumour, nodal, metastasis) classification, time-point of metastatic disease (synchronous versus metachronous), size and spread of liver metastases, RAS mutational status, extent of liver resection: minor vs. major (3 or more liver segments or ≥6 atypical resections/ablations). Ninety-days postoperative morbidity and mortality were recorded and classified according to the Clavien-Dindo (C-D) classification [37] and grouped as mild (C-D I-IIIa) and severe (C-D IIIb-V). The study protocol was approved by the medical ethics committees of both centres (Protocol-number Salzburg: 415-EP/73/629-2016 and Innsbruck: 1033/2017), waiving the need for written informed consent due to the retrospective design.

OS was defined as the time from hepatic resection until the date of death or the last date of follow up (censored) and was crosschecked with national data from the Statistics Austria death registry [38]. DFS was defined as the time from initial clearance of all tumour deposits (primary, hepatic and extrahepatic) to first recurrence at any site. Death from other non-CRC specific causes was not defined as an event in DFS analysis (censored). The results are presented according to the “Strengthening the reporting of observational studies in epidemiology” (STROBE) checklist for cohort studies [39].

#### Statistical analysis

Patient and tumour characteristics are presented as numbers and associated percentages for categorical data and as mean and standard deviation for continuous variables. Differences between patients with right- and left-sided primary tumour were analysed with the Chi-square test or Fisher’s Exact test for categorical and the Mann-Whitney U for continuous variables. Distribution of normality was tested using the Shapiro-Wilk test. Survival curves were plotted by the Kaplan Meier method, and survival differences between sidedness groups were calculated by the log-rank test. OS values are estimates according to Kaplan-Meier method. To identify predictors of survival, univariable and multivariable analysis was performed using Cox proportional hazards regression models. All variables with p≤0.10 in univariable analysis were entered into the multivariable model after exclusion of multicollinearity. A clinical risk score for prediction of survival was created through non-time-dependent receiver operating characteristics (ROC) curves and area under the curve (AUC) analysis to identify the predictive value of single factors for death during follow-up. Estimation of time-dependent AUC analysis with 95% confidence intervals of this score was performed using the timeROC package in R, which creates a time-dependent ROC curve from censored survival data, as previously described by Blanche et al. [40]. Compared to the classical approach for ROC curve analysis that considers event status and marker value for an individual as fixed over time, time-dependent ROC curve analysis computes AUC values dynamically over time for each given time-point (e.g. 12 months, 60 months) and different markers variables (e.g. risk-score groups) [41].

A p-value <0.05 was considered statistically significant throughout all tests. Statistical analysis was performed with IBM SPSS software (version 24.0; IBM Inc., USA) and R (www.r-project.org).

#### International validation cohort

The results of our own bi-institutional experience were validated with data on patients undergoing liver resection in two international hepatobiliary centres: The Aintree University Hospital in Liverpool, United Kingdom provided data of 364 patients from 2010 to 2015, and the Kumamoto University in Japan participated with 163 patients operated between 2005 and 2016. Details on inclusion criteria and characteristics of these cohorts have previously been published [15, 42]. Essentially, the inclusion criteria were similar to our own cohort. Statistical analysis involved the methods described above.

### Meta-analysis

#### Literature search

PUBMED and OVID were searched in October 2018 for literature evaluating primary tumour location in the context of liver resection for CRLM, with no limitation in terms of publication time-period. The MeSH terms “colorectal cancer”, “colon cancer”, “colonic neoplasm” or “colorectal neoplasm” and “primary tumour location” or “primary tumor location” or “embryonic origin” or “sidedness” were combined with “liver resection” or “hepatectomy” and “survival”.

#### Inclusion and exclusion criteria

The meta-analysis was conducted according to the Preferred Reporting Items for Systematic Reviews and Meta-analyses (PRISMA) guidelines [43]. The primary end point was association of primary tumour sidedness with OS and DFS after liver resection. Studies needed to fulfil the following criteria to be included in the meta-analysis 1) Availability of data on OS or DFS after CRLM resection and information about primary tumour location; 2) Hazard ratio (HR) and 95% confidence interval (CI) for OS or DFS according to primary tumour site was reported in the study 3) the prognostic effect was determined as a function of the mortality of the patients, and the follow-up period was at least 2 years. The exclusion criteria were as follows: 1) letters, reviews, case reports, conference abstracts (except ASCO congress abstracts, which are comparable to full studies), editorials, and expert opinions; 2) articles in which no information on OS / DFS was given or the HR could not be calculated from the given information. All retrieved results in English language were screened by reading of the title and abstract and after filtering by reading the full paper by two reviewers (E.G. and F.P.). If data from the same study cohort had been published repeatedly, the most relevant publication was chosen and included in the analysis only once [44, 45].

#### Statistical analysis

MedCalc Statistical Software version 18 (MedCalc Software, Ostend, Belgium) and Review Manager 5.3 (The Nordic Cochrane Centre, Rigshospitalet, Copenhagen, Denmark) was used to perform the meta-analysis with the generic inverse variance method. Study-specific HRs and 95% CIs for OS and DFS were extracted from literature as described above and summarized with both the fixed and random effects model. The impact of sidedness (RCRC) on worse OS / DFS was considered significant, when the 95%CI for combined effect did not cross the HR value of 1 (analogous to a p-value <0.05). The *I*^2^ statistics according to Higgins et al. was used to evaluate for heterogeneity among included studies [46]. The inconsistency (extent of heterogeneity) was defined as the following: *I*^2^<25% = no heterogeneity; *I*^2^25-50% = moderate heterogeneity; *I*^2^50-75% = high heterogeneity; *I*^2^>75% = severe heterogeneity [47, 48]. Individual and summarized effect estimates were presented graphically with a forest plot. To evaluate for potential publication and small study bias, a funnel plot was generated [49]. All calculations were first done for studies including colon and/or rectal cancer patients, subsequently a sub-analysis of only those studies that excluded rectal cancer patients was performed, as well sub-analysis according to geographical region of included studies.

## Results

### Own cohort data

#### Patients characteristics

A total of 259 patients underwent liver resection for newly diagnosed CRLM and were analysed. In terms of primary tumour sidedness, 59 patients (22.8%) had a RCRC while 200 (77.2%) had a LCRC, including 95 (36.7%) rectal cancer patients. The exact primary tumour site included caecum (n = 20; 7.7%), ascending colon (n = 25; 9.7%), hepatic flexure (n = 6; 2.3%), transverse colon (n = 8; 3.1%), splenic flexure (n = 3; 1.2%), descending colon (n = 8; 3.1%), sigmoid colon (n = 94; 36.3%) and rectum (n = 95; 36.7%).

#### Differences between right- and left-sided primary tumour patients

A comparison of patients’ primary tumour and metastases characteristics according to sidedness is provided in Table 1. Patients with RCRC were older than patients with LCRC (66.2 vs. 64.3 years), although not statistically significant (p = 0.067). There was no significant difference between RCRC and LCRC patients in terms of female-to-male ratio, median BMI and ASA status. The majority of both RCRC and LCRC patients had advanced (T3/T4) primary tumour stage. Pathological examination revealed a significantly higher percentage of positive lymph node involvement in 76.3% vs. 61.3% (p = 0.043) in RCRC, and more RAS mutations (60% vs. 34.9%; p = 0.036).

**Table 1:** Patient and tumour characteristics according to primary tumour sidedness.

Characteristics	Right-sided (n = 59)	Left-sided (n = 200)	p-value
Age (median, Range; years) *	66.2 (36.1–87.8)	64.3 (32.9–83.5)	0.067
Sex			0.369
male	39 (66.1%)	119 (59.5%)	
female	20 (33.9%)	81 (40.5%)	
BMI (median, IQR, kg/m^2^)	24.8 (16.3–37.4)	24.2 (16.1–40.4)	0.999
ASA status			0.887
1–2	31/45 (68.9%)	103/152 (67.8%)	
3–4	14/45 (31.1%)	49/153 (32.2%)	
T stage primary tumour			0.078
T1–2	2/59 (3.4%)	22/193 (11.4%)	
T3–4	57/59 (96.6%)	171/193 (88.6%)	
Nodal metastases for primary tumour			0.043
positive	45/59 (76.3%)	122/199 (61.3%)	
negative	14/59 (23.7%)	77/199 (38.7%)	
Grading for primary tumour			0.056
1	0/57(0%)	9/183 (4.9%)	
2	37/57 (64.9%)	132/183 (72.1%)	
3	20/57 (35.1%)	42/183 (23%)	
RAS status *(missing = 148)*			0.036
wild-type	10/25 (40%)	56/86 (65.1%)	
mutated	15/25 (60%)	30/86 (34.9%)	
Presentation of liver metastases (<6 months)			0.351
synchronous	35 (59.3%)	134 (67%)	
metachronous	24 (40.7%)	66 (33%)	
Bilobar disease			0.447
yes	25 (42.4%)	73 (36.5)	
no	34 (57.6%)	127 (63.5%)	
Extrahepatic disease	6 (10.2%)	17 (8.5%)	0.794
Number of liver metastases			0.545
1	21 (35.6%)	82 (41%)	
>1	38 (64.4%)	118 (59%)	
Size of liver metastases			0.570
≤ 5 cm	50 (84.7%)	162 (81%)	
>5 cm	9 (15.3%)	38 (19%)	
Preoperative chemotherapy	27/59 (45.8%)	113/200 (56.5%)	0.181
Oxaliplatin ***(missing = 1)***	24/26 (92.3%)	84/113 (74.3%)	0.065
Irinotecan ***(missing = 1)***	4/26 (15.4%)	37/113 (32.7%)	0.097
Anti-EGFR ***(missing = 4)***	4/26 (15.4%)	34/110 (30.9%)	0.147
Bevacizumab ***(missing = 1)***	7/26 (26.9%)	40/113 (35.4%)	0.495
Liver resection extent			0.524
minor	43 (72.9%)	136 (68%)	
major	16 (27.1%)	64 (32%)	

ASA = American Society of Anesthesiology; BMI = Body mass index; cm = centimetre; EGFR = Epidermal growth factor receptor; IQR = interquartile range

*at date of liver resection

Regarding characteristics of metastatic extent as well as surgical and systemic treatment, there was no significant difference between the two groups: Most patients presented with synchronous disease (RCRC: n = 35, 59.3%; LCRC: n = 134, 67%; p = 0.351) and multiple liver metastasis (RCRC: n = 38, 64.4%; LCRC: n = 118, 59%; p = 0.545), bilobar involvement was present in 42.4% and 36.5%, respectively (p = 0.447). Up to 10 per cent in both groups had an additional extrahepatic lesion at the time of diagnosis of liver metastasis (p = 0.794), and almost every fifth patient had a liver lesion larger than 5 cm (p = 0.570). Concerning treatment, the majority of patients underwent minor resection (RCRC: n = 43, 72.9%; LCRC: n = 136, 68%; p = 0.524). The resulting 90-days mortality was 5.1% vs 0.5% (RCRC: n = 3; LCRC: n = 1; p = 0.038), whereby two deaths in the RCRC-group occurred owing to simultaneous primary tumour surgery complications (one anastomotic insufficiency with sepsis and one paralytic ileus with aspiration after right colectomy). Without these two cases, resulting liver-surgery specific 90-days mortality was 1.8% (RCRC) vs. 0.5% (LCRC; p = 0.395). The overall 90-days morbidity was 37.3% vs 29% (RCRC: n = 22; LCRC: n = 58; p = 0.262) and 90-days severe morbidity 17% (RCRC: n = 10) vs 10.5% (LCRC: n = 21; p = 0.252). Sub-analysis of characteristics comparing left-sided and right-sided colon cancer and rectal cancer showed no significant differences regarding age (p = 0.176), sex (p = 0.186), BMI (p = 0.149), ASA status (p = 0.975), T-stage (p = 0.135), nodal status (p = 0.105), timing of metastases (p = 0.490), bilobar distribution (p = 0.528), EHD (p = 0.320), number of LM (p = 0.723), size of LM (p = 0.068), application of preoperative CTX (p = 0.279) and extent of resection (p = 0.451). However, grading (p = 0.045) and RAS-status (p = 0.048) was significantly different, with right-sided CRC showing the highest rate of G3 tumours (35.1%) and RAS mutations (60%).

#### Oncological outcome and factors influencing survival

**Fig 1:**
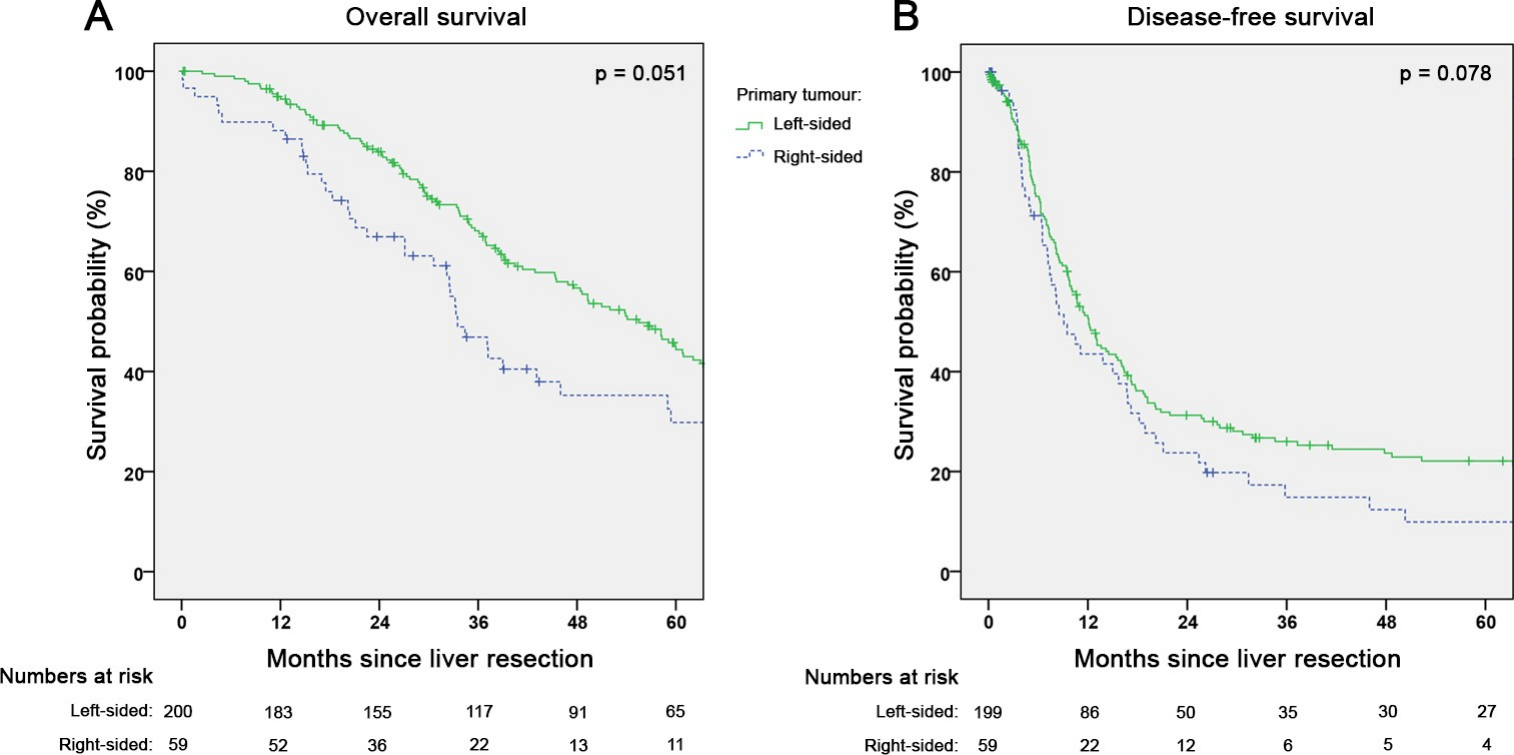
Survival according to primary tumour sidedness after hepatectomy for colorectal liver metastases. (A) Overall survival (n = 259; p = 0.051) and (B) disease-free survival (n = 258; missing = 1; p = 0.078) in own cohort of patients undergoing curative-intent liver resection for colorectal liver metastases.

The median follow-up regarding OS was 38.1 months (0.1–157.3). The median OS after CRLM resection was 49.3 months in all patients (95%CI 40.3–58.3), 33.5 months (95%CI 27.6–39.4) in RCRC versus 55.5 months (95%CI 47.8–63.2) in LCRC (p = 0.051; Fig 1A). The median DFS (missing n = 1) was 11.4 months (95%CI 9.4–13.4) with a recurrence rate of 70.7% within the study follow-up period, resulting in a 5-year recurrence-free survival rate of 19.2%. The median DFS for RCRC was 9.1 months (95%CI 5.6–12.6) and 12.1 months for LCRC (95%CI 10.0–14.2; p = 0.078; Fig 1B). OS stratified by right-sided versus left-sided colon cancer and rectal cancer, revealed no significant difference between left-sided colon and rectal cancer patients with 55.5 months (95%CI 45.9–65.1) and 58.2 months (95%CI 44.6–71.8), respectively. The OS for right-sided colon cancer patients was markedly worse (33.5 months; 95% CI 27.6–39.4), however not statistically significant compared to rectal (p = 0.058) and left-sided colon cancer p = 0.125) patients. The DFS for right-sided colon cancer also was the worst among the three groups with 9.1 months (95%CI 5.6–12.6), statistically different from left-sided colon cancer patients (12.9 months; 95%CI 9.0–16.8; p = 0.029) but not rectal cancer cases (9.6 months; 95%CI 5.4–13.8; p = 0.344).

Univariable analysis of factors associated with OS after resection of CRLM (Table 2) revealed age >60 years (HR 0.67, 95%CI 0.43–1.03), nodal status (HR 1.64, 95%CI 1.16–2.31), right-sided primary tumour location (HR 1.44, 95%CI 0.99–2.08), EHD (HR 1.78, 95%CI 1.08–2.96) and hepatic bilobar involvement (HR 1.54, 95% CI1.11–2.14) as variables with a p-value ≤0.1. When incorporated in a multivariable model, nodal status (HR 1.52, 95%CI 1.05–2.19; p = 0.026), right-sided primary tumour location (HR 1.53, 95%CI 1.04–2.25; p = 0.029), EHD (HR 1.71, 95%CI 1.02–2.85; p = 0.041) and hepatic bilobar involvement (HR 1.41, 95%CI 1.00–1.99; p = 0.048) were significantly associated with worse OS. Sub-analysis excluding the two aforementioned RCRC cases with non-liver-surgery specific 90-days-mortality resulted in the following hazard ratios: nodal status: 1.49 (95%CI 1.03–2.16; p = 0.033); right-sided primary tumour location: 1.45 (0.98–2.15; p = 0.061); EHD: 1.73 (1.03–2.9; p = 0.037), hepatic bilobar disease: 1.46 (1.03–2.06; p = 0.031).

**Table 2:** Factors associated with survival after resection of colorectal liver metastases.

	Univariable (OS)			Multivariable (OS)			Univariable (DFS)			Multivariable (DFS)		
	HR	95% CI	p	HR	95% CI	p	HR	95% CI	p	HR	95% CI	p
Age (years)*												
≤60		Ref.			Ref.			Ref.			Ref.	
>60	0.67	(0.43–1.03)	0.065	0.71	(0.50–1.00)	0.050	0.76	(0.56–1.03)	0.072	0.77	(0.57–1.06)	0.106
Sex												
male		Ref.						Ref.				
female	1.00	(0.73–1.38)	0.995				0.90	(0.67–1.22)	0.509			
ASA												
1–2		Ref.						Ref.				
3–4	1.31	(0.90–1.91)	0.163				1.10	(0.78–1.57)	0.582			
T stage												
1–2		Ref.			Ref.			Ref.				
3–4	1.80	(0.98–3.33)	0.060	1.50	(0.80–2.80)	0.208	1.27	(0.75–2.16)	0.374			
Nodal metastases primary tumour												
Nodal negative		Ref.			Ref.			Ref.			Ref.	
Nodal positive	1.64	(1.16–2.31)	0.005	1.52	(1.05–2.19)	0.026	1.47	(1.08–2.01)	0.015	1.34	(0.97–1.84)	0.073
Grading primary tumour												
G1-G2		Ref.						Ref.				
G3	1.31	(0.91–1.88)	0.150				1.13	(0.82–1.58)	0.452			
Primary tumour location												
Left		Ref.			Ref.			Ref.			Ref.	
Right	1.44	(0.99–2.08)	0.052	1.53	(1.04–2.25)	0.029	1.35	(0.97–1.88)	0.080	1.33	(0.95–1.87)	0.099
Extrahepatic disease												
No		Ref.			Ref.			Ref.			Ref.	
Yes	1.78	(1.08–2.96)	0.025	1.71	(1.02–2.85)	0.041	1.91	(1.21–3.03)	0.006	1.62	(1.01–2.60)	0.046
Presentation of liver metastases												
synchronous		Ref.						Ref.				
metachronous	1.02	(0.74–1.42)	0.888				0.99	(0.73–1.34)	0.926			
Number of liver metastases												
1		Ref.						Ref.				
>1	1.31	(0.95–1.80)	0.103				1.27	(0.94–1.72)	0.124			
Size of liver metastases												
≤5 cm		Ref.						Ref.				
>5 cm	1.01	(0.68–1.51)	0.952				0.98	(0.64–1.44)	0.923			
Bilobar disease												
No		Ref.			Ref.			Ref.			Ref.	
yes	1.54	(1.11–2.14)	0.009	1.41	(1.00–1.99)	0.048	1.62	(1.20–2.20)	0.002	1.42	(1.03–1.95)	0.031
RAS Mutation												
no		Ref.						Ref.				
yes	1.21	(0.73–2.01)	0.455				0.86	(0.56–1.33)	0.504			
Preoperative Chemotherapy												
yes		Ref.						Ref.				
no	1.25	(0.92–1.72)	0.156				1.22	(0.91–1.63)	0.190			
Liver resection												
minor		Ref.						Ref.				
major	1.30	(0.94–1.82)	0.117				1.03	(0.75–1.42)	0.854			

Left: Factors associated with overall survival (OS) in univariable and multivariable cox-regression analysis. Right: Factors associated with disease-free survival (DFS) in univariable and multivariable cox-regression analysis. ASA = American Society of Anesthesiology; CI = confidence interval; cm = centimetre; HR = Hazard ratio

*at date of liver surgery

Regarding DFS, univariable analysis showed that age >60 years (HR 0.76, 95%CI 0.56–1.03), nodal status (HR 1.47, 95%CI 1.08–2.01), sidedness (HR 1.35, 95% CI 0.97–1.88), EHD (HR 1.91, 95% CI 1.21–3.03) and bilobar disease (HR 1.62, 95%CI 1.20–2.20) were associated with recurrence (p≤0.1). The multivariable model including these factors revealed, that only EHD (1.62; 95%CI 1.01–2.60; p = 0.046) and bilobar disease (HR 1.42, 95%CI 1.03–1.95; p = 0.031) remained statistically significant. Sidedness was not independently associated with DFS (HR 1.33, 95%CI 0.95–1.87; p = 0.099).

#### Applicability of sidedness as a factor in clinical risk scores

First, the ability of single clinical risk factors to predict death during follow-up in our cohort were evaluated using non-time-dependent ROC analysis. Primary tumour lymph node positivity (AUC 0.579) and EHD (AUC 0.523) showed best discrimination, followed by number of liver metastases (>1 CRLM, AUC: 0.518) and sidedness (RCRC, AUC: 0.514), whereas size of CRLM (>5cm, AUC 0.501) and bilobar disease (AUC 0.499) were not predictive. Consequently, the first four factors were combined in different clinical risk score models regarding OS and DFS after resection for CRLM. After evaluation by Kaplan-Meier and time-dependent ROC analysis, the final clinical risk score model comprised of three factors: primary tumour sidedness, lymph node status and presence of EHD. Each predictive factor (RCRC, nodal positivity and EHD) was assigned one point, resulting in a score between 0 and 3 points.

**Fig 2:**
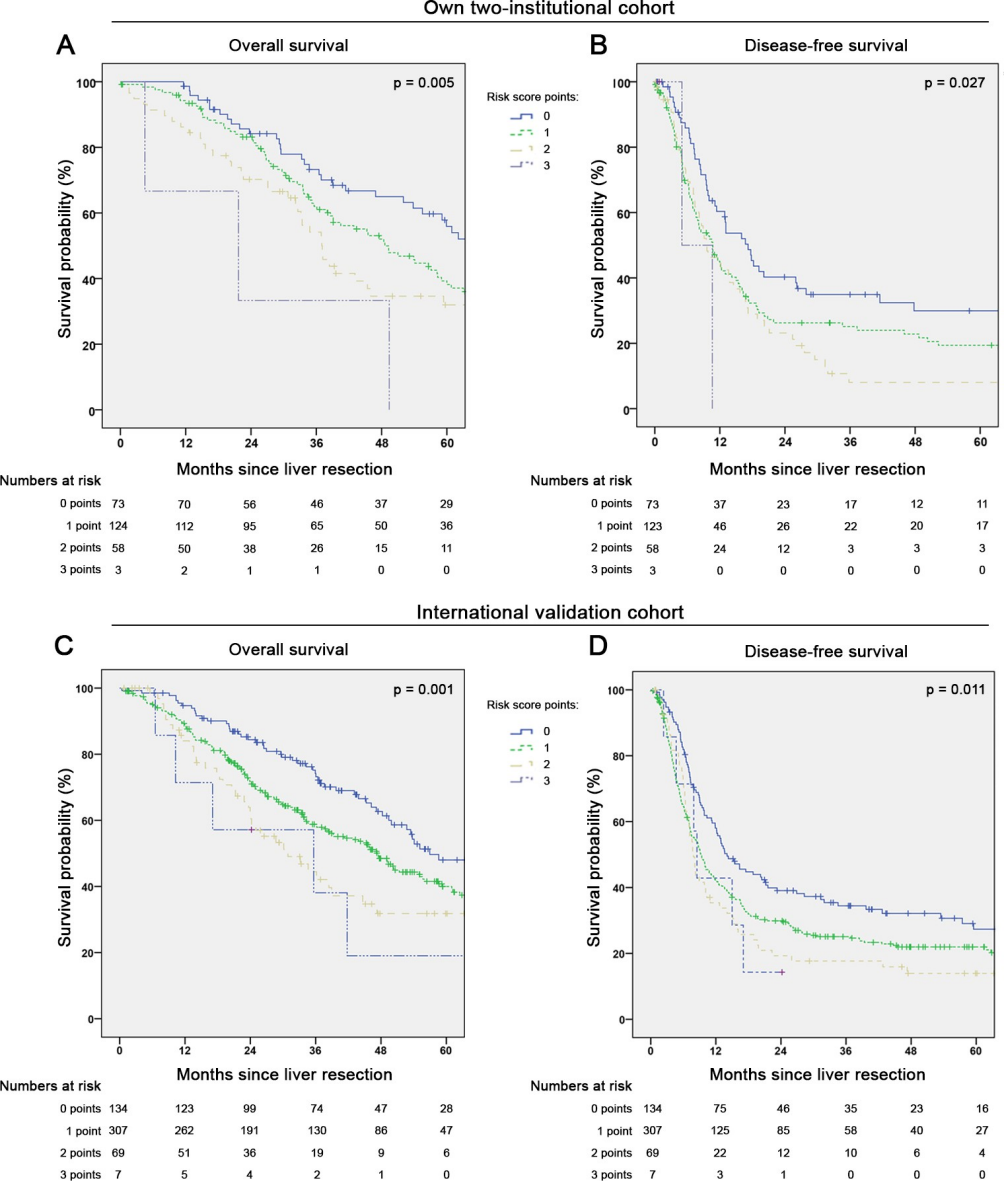
Survival according to a clinical risk score including sidedness, nodal positivity and extrahepatic disease. (A) Overall survival (n = 258; missing = 1; p = 0.005) and (B) disease-free survival (n = 257; missing = 2; p = 0.027) in own cohort of patients undergoing curative-intent liver resection for colorectal liver metastases. Each factor (right-sided primary-tumour, positive lymph-nodes and extrahepatic disease) was accounted for one point in this score). (C) Overall survival (n = 517; missing = 10; p = 0.001) and (D) disease-free survival (n = 517; missing = 10; p = 0.011) in the international validation cohort.

This clinical risk score provided significant survival discrimination between the groups (p = 0.005) as depicted in Fig 2A. The median OS for patients without any of these three risk factors (0 points; n = 73) was 66.7 months (95%CI 54.0–79.4), compared to 48.6 months (95%CI 38.4–58.8) for patients with 1 point (n = 124), 37.0 months (95%CI 31.6–42.4) for two points (n = 58) and 21.7 months (0.0–49.2) for three points (n = 3). The DFS (Fig 2B) was also significantly different (p = 0.027) between the risk groups: 0 points 17.2 months (95%CI 12.4–22.0), 1 point 10.7 months (95%CI 7.5–13.9), 2 points 9.6 months (95%CI 5.5–13.7) and 3 points 5 months (95%CI not applicable). The time-dependent ROC analysis estimated an AUC for OS of 0.701 (95%CI 0.592–0.810) at 12 months and 0.615 (95%CI 0.542–0.688) at 60 months. Regarding DFS the estimated time-dependent AUC was 0.561 (95%CI 0.491–0.632) at 12 months and 0.610 (95%CI 0.520–0.700) at 60 months.

#### International validation of the clinical risk score

The validation cohort included a total of 527 patients with characteristics comparable to our own cohort in terms of age >60 (70.8%; p = 0.175), primary tumour nodal positivity (60.9%; p = 0.304), T-stage (T3/T4: 85.7%; p = 0.063) and sidedness (RCRC: 20.9%; p = 0.541), hepatic bilobar involvement (42%; p = 0.398) and presence of EHD (8.3%; p = 0.802). In the validation cohort, the median OS and DFS after surgery were 47.3 months (95%CI 41.7–52.9) and 10.0 months (95%CI 8.5–11.4), also both comparable our own cohort (p = 0.544 and p = 0.936, respectively). Validation multivariable analysis of factors associated with survival in univariable analysis confirmed the previous findings from our own patients: Nodal positivity (HR 1.44; 95%CI 1.11–1.88; p = 0.006), presence of EHD (HR 1.58; 95%CI 1.05–2.37; p = 0.029) and RCRC (HR 1.42; 95%CI 1.05–1.92; p = 0.022) were independently linked to worse OS. Regarding DFS, only nodal positivity (HR 1.33; 95%CI 1.08–1.65; p = 0.007) and presences of EHD (HR 1.56; 95%CI 1.11–2.19; p = 0.010) was associated with worse outcome in multivariable analysis, while sidedness was not (RCRC: HR 1.08; 95%CI 0.84–1.39; p = 0.565). In both OS and DFS analysis, age>60 was not independently linked to outcome and bilobar distribution was not included, due to missing data in one centre.

In a total of 517 validation patients, all 3 variables for the proposed clinical risk factor were recorded. Distribution of risk-point assignment according to the score showed minor differences between the own and the validation cohort: 0 points: 28.3% vs. 25.9%; 1 point: 48.1% vs. 59.4%; 2 points: 22.5% vs. 13.3%; 3 points: 1.2% vs. 1.4%; p = 0.004). Survival curves according to the risk score in the validation set are provided in Fig 2C and 2D. The score was significantly associated with OS (p = 0.001) and DFS (p = 0.011), however with limited discrimination between patients with 2 and 3 risk factors: The median OS for patients with 0 points was 57.0 months (95%CI 36.1–77.8), 47.3 months (95%CI 40.5–54.1) for 1 point, 30.9 months (95%CI 20.4–41.4) for 2 points and 35.6 months (95%CI 0.0–73.7) for 3 points. The median DFS was 14.1 months (95%CI 10.0–18.1) for patients with 0 points, 9.3 months (95%CI 7.8–10.9) for 1 point, 7.9 months (95%CI 6.7–9.1) for 2 points and 8.5 months (95%CI 7.0–10.0) for 3 points.

### Meta-analysis

#### Publication selection and baseline study characteristics

**Fig 3:**
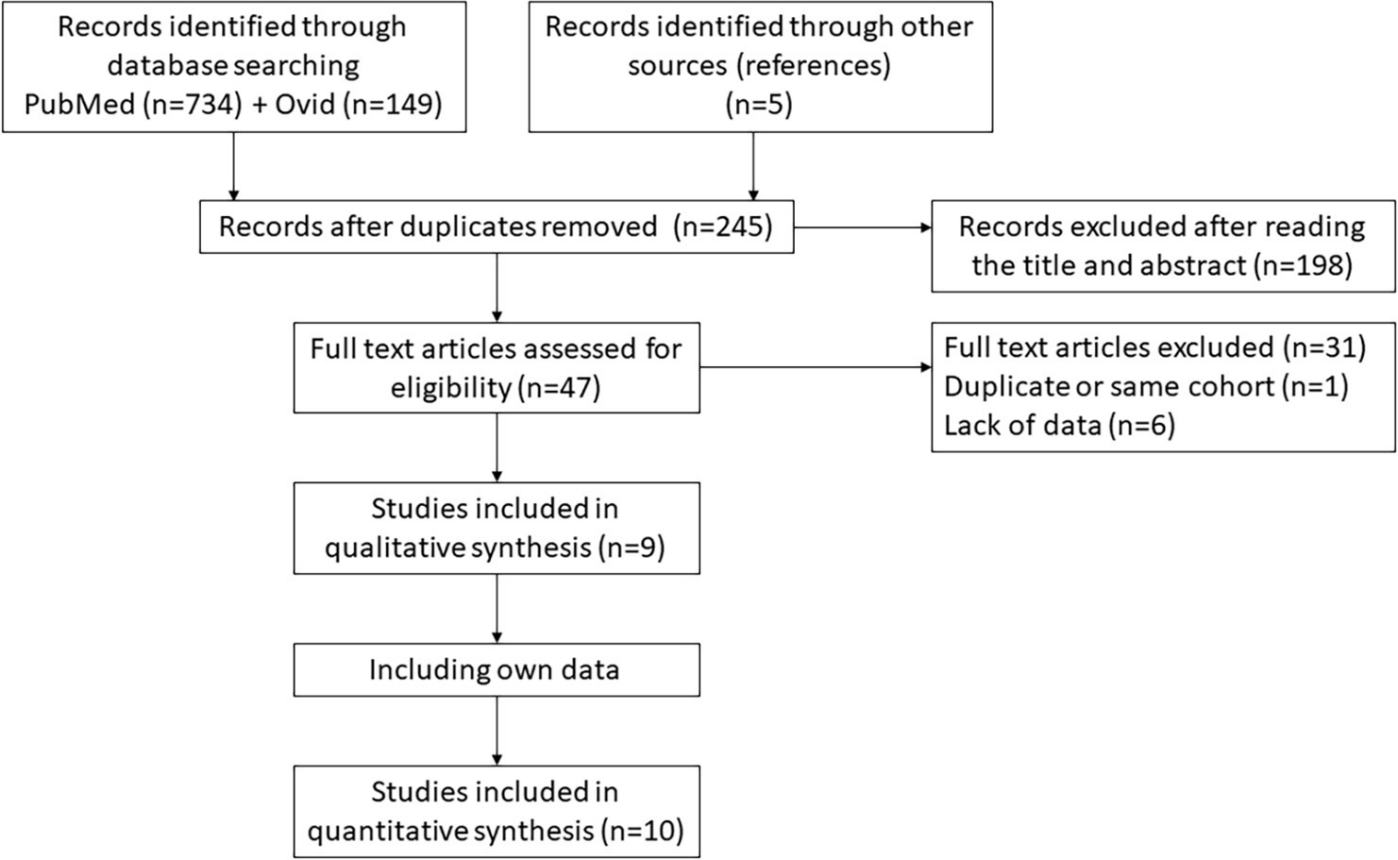
CONSORT diagram of study selection for meta-analysis on sidedness and survival after liver resection. Literature search initially revealed 883 studies of which 9 were included in the final analysis (10 including own data).

Fig 3 demonstrates the CONSORT flow diagram of the literature search and study selection process. The systematic search initially resulted in an output of 883 studies, of which 9 were included in the final meta-analysis (all were retrospective cohort studies). The characteristics and outcomes of these studies are summarized in Table 3. Four studies excluded rectal cancer patients in their analysis. Including our own cohort, a total of 4,312 patients (RCRC: n = 1493; LCRC: n = 2873), ranging from 72 to 907 patients per cohort were included. HR and 95%CI for OS could be retrieved from all 9 studies, whereas DFS was only available in 5 of these reports. The median total follow-up for OS ranged from 26 to 42 months.

**Table 3:** Studies included in meta-analysis regarding primary tumour location and survival after liver metastases resection.

Study	Year	Study period	Number of patients	Sidedness: R/L primary	Sex (m/f)	OS: HR (95%CI)	p-value	DFS: HR (95%CI)	p-value	Demarcation line R/L; notes	Rectum included	Median follow up (total—in months)
Sasaki et al. [44]	2016	2003–2015	475	191/284	270/205	1.41 (1.03–1.96)	0.033	0.75 (0.57–1.0)	0.049	splenic flexure	yes	30.7
Lee-Ying et al. [50]	2017	2004–2016	471	204/267	297/174	1.4 (1.0–1.9)	0.02	n.a.	n.a.	splenic flexure	no	n.a.
Marques et al. [51]	2018	1998–2012								splenic flexure	yes	42
- KRAS^mut^			28	9/19	n.a.	2.1 (0.5–8.3)	0.281	1.1 (0.5–2.5)	0.785			
- KRAS^wt^			63	3/60	n.a.	1.1 (0.4–2.6)	0.877	1.0 (0.5–1.8)	0.971			
Creasy et al. [16]	2018	1992–2004	907	329/578	508/399	1.22 (1.02–1.45)	0.028	1.14 (0.97–1.35)	0.105	splenic flexure	no	n.a.
Dupré et al. [15]	2018	2010–2015	364	74/290	250/114	1.90 (1.23–2.94)	0.004	n.a.	n.a.	splenic flexure	yes	41.8
Wang et al. [14]	2018	2002–2015	420	86/334	257/163	1.08 (0.76–1.53)	0.655	n.a.	n.a.	splenic flexure	yes	26
Yamashita et al. [36]	2018	1990–2015								transverse excl.	no	27
- with preop. CTX			725	238/487	422/303	2.04 (1.60–2.59)	<0.0001	1.71 (1.41–2.07)	<0.0001			
- without preop. CTX			252	89/163	n.a.	1.90 (1.29–2.77)	0.0009	1.48 (1.05–2.08)	0.026			
Goto et al. [52]	2018	2004–2015	276	138/138	n.a.	1.79 (1.26–2.5)	<0.01	1.29 (0.82–2.05)	0.27	splenic flexure	no	n.a.
Imai et al. (no CTX) [42]	2018	2005–2016	72	19/53	n.a.	3.44 (1.21–10.03)	0.021	n.a.	n.a.	splenic flexure	yes	38.8
Own data	2019	2003–2016	259	59/200	158/101	1.53 (1.04–2.25)	0.029	1.33 (0.95–1.87)	0.099	splenic flexure	yes	38.1

CI = confidence interval; DFS = disease-free survival; f = female; HR = hazard ratio; KRAS^mut/wt^ = KRAS mutated or wild-type subgroup; m = male; n.a. = data not available; OS = overall survival; preop. CTX = preoperative chemotherapy; R/L = right or left-sided primary tumour; transverse excl. = transverse colon was excluded in this study.

#### Association of sidedness with survival

**Fig 4:**
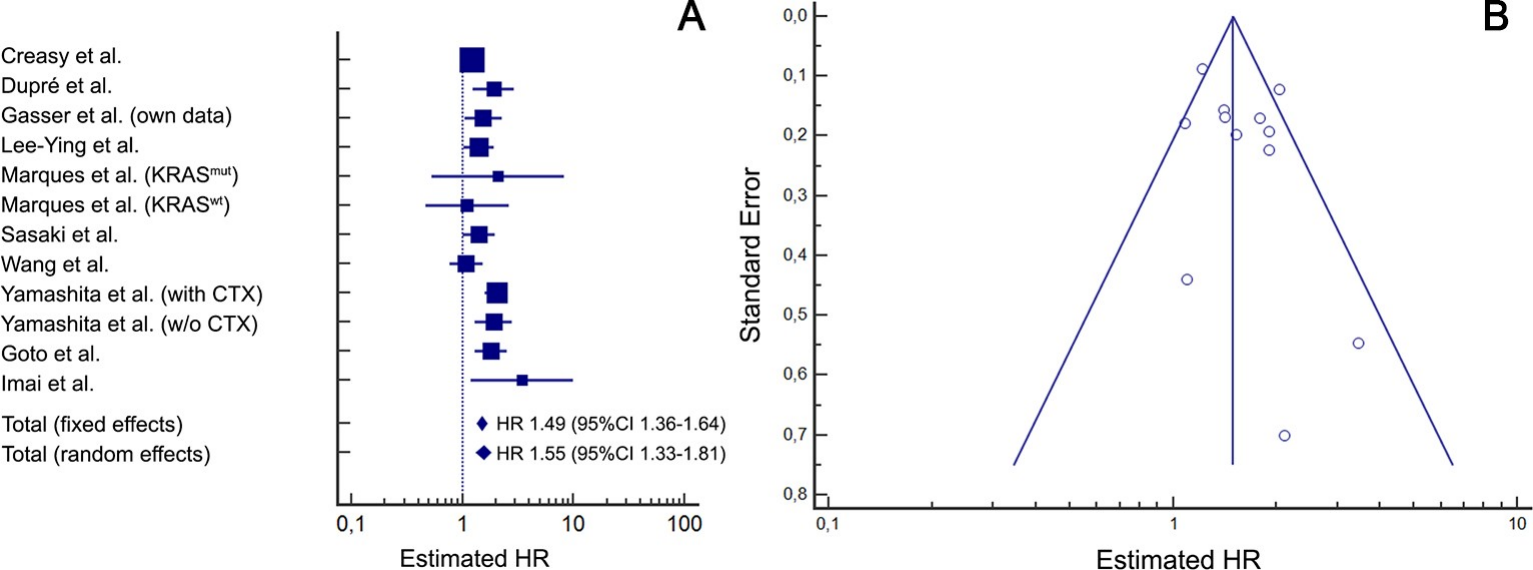
Association of right-sided primary tumour location with overall survival (meta-analysis). Meta-analysis of published studies including own data (Heterogeneity: *I*^2^ = 50.8% / p = 0.0216). (A) Forrest plot: right-sided primary tumour location is significantly associated with worse overall survival after resection for liver metastases (random effects model: p<0.001) (B) Funnel plot analysis does not indicate a relevant publication bias but minor small trial bias: While the number of larger studies (tip of the pyramid) on both sides of the total effects line are evenly distributed, two smaller studies with an overestimating positive effect have only one negative study as counterparts (base of the pyramid). CI = confidence interval; HR = hazard ratio; KRAS^mut/wt^ = KRAS mutated or wild-type subgroup; with or w/o CTX = with or without preoperative chemotherapy.

**Fig 5:**
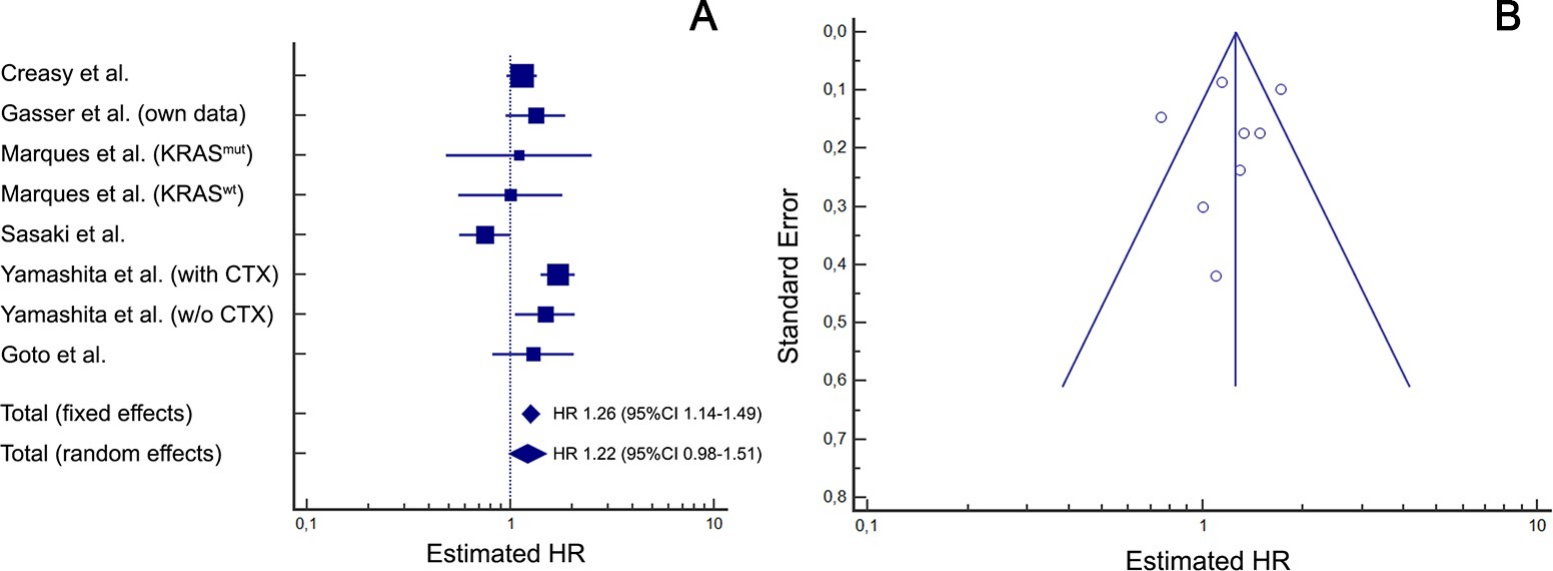
Association of right-sided primary tumour location with disease-free survival (meta-analysis). Meta-analysis of published studies including own data (Heterogeneity: *I*^2^ = 72.4% / p<0.001). (A) Forrest plot: right-sided primary tumour location is associated with worse disease-free survival after resection for liver metastases, however not significant in the random effects model (p = 0.077). (B) Funnel plot analysis does indicate only a minimal publication bias with two large study outliers outside the 95%CI (at the tip of the pyramid) but no small trial bias (two smaller studies on the left side of the overall effects line have larger counterparts on the right side). CI = confidence interval; HR = hazard ratio; KRAS^mut/wt^ = KRAS mutated or wild-type subgroup; with / w/o CTX = with or without preoperative chemotherapy.

In the meta-analysis evaluating the impact of sidedness on OS after resection of CRLM (Fig 4A) moderate to high heterogeneity (*I*^2^ = 50.8%) was observed amongst included studies. Hence, for final interpretation the random-effects model was considered, in which right-sided primary tumour location was significantly associated with worse OS (HR 1.55; 95%CI 1.33–1.81; p<0.001). Funnel plot asymmetry analysis (Fig 4B) showed a symmetrical distribution with no indication for significant publication bias but suggests minor small study bias. Regarding DFS (Fig 5A), the heterogeneity among the 5 included studies was high (*I*^2^ = 72.4%), and the resulting random-effects model showed a HR of 1.22 (95%CI 0.98–1.51; p = 0.077). The associated funnel plot (Fig 5B) showed no distinct asymmetry despite two larger study outliers, suggesting evidence for only a minimal publication bias and no relevant small study bias. According to previous recommendations, statistical testing for funnel plot asymmetry (e.g. with Egger’s test) was not performed due to the limited number of studies available for this meta-analysis [53].

Following the ongoing discussion, whether rectal cancer patients should be included in the left-sided group, we also performed a sub-analysis excluding all studies with rectal cancer. This did not result in a significantly different heterogeneity regarding OS and DFS. The HR of RCRC was 1.62 (95%CI 1.28–2.04; p<0.001) for OS and 1.39 (95%CI 1.10–1.76; p = 0.006) for DFS in this sub-analysis. Detailed data including study weights of all different meta-analysis models are provided as supplement (S1–S4 Tables)

Furthermore, we aimed to perform sub-analysis of included studies according to racial disparities. However, none of the evaluated manuscripts provided information on patient race. Assuming, that the vast majority of patients from Asian centres are Asians, from European centres are Caucasians and American centres have mixed ethnicities of Caucasians, African American and Latin American patients, geographic regions may be used as surrogates. Therefore, a geographical stratification was conducted, comparing all studies from North or South America with Europe and Asia. Results are shown in S1 Fig and S2 Fig. Regarding OS, there was a statistically significant association with RCRC in the five studies with American cohorts (HR 1.53; 95%CI 1.25–1.87) and three European studies (HR 1.73; 95%CI 1.39–2.15), but not in the two Asian cohorts (HR 1.72; 95%CI 0.56–5.23). Concerning DFS, no Asian study reported outcomes. In the four American studies with available results, no significant association of RCRC with worse DFS could be found (HR 1.18; 95%CI 0.89–1.56), while results from the two European cohorts closely achieved statistical significance (HR 1.32; 95%CI 1.00–1.73, respectively).

## Discussion

The present study examined if survival after resection of CRLM is worse in patients with RCRC compared to LCRC through analysis of own data, international validation cohorts and meta-analysis of published studies. First, we evaluated 259 patients undergoing liver surgery in two Austrian University Hospitals, revealing differences in biological behaviour between RCRC and LCRC tumours. RCRC significantly more often had positive lymph nodes and RAS mutations, and patients with RCRC tended to be older than LCRC patients’, which is in line with other studies [15, 54]. In contrast to previous reports, a sex difference with higher incidence of RCRC in women was not seen in our own data [14, 54, 55].

In terms of survival data validity, the present study substantially differs from other previous publications on this specific topic. Despite retrospective analysis, our data are based on an auditable, prospectively maintained database with incorporation of national death statistics, potentially strengthening our results. Our median follow-up was 38.1 months, which is comparably longer than in a number of other studies (Table 3). [12–16]. Regarding short-term postoperative outcome, RCRC showed increased 90-days-mortality due to simultaneous colon-surgery related deaths (one sepsis due to anastomotic leakage and one paralytic ileus with aspiration). It has recently been reported by others, that ileo-colic and colo-colic anastomosis might possess an increased risk of anastomotic leakage [56]. On the contrary, in our cohort liver-surgery related mortality was not significantly different between the two groups. To account for these influences, multivariable analysis was performed first with and then without these two cases of colon-surgery related postoperative death. The first analysis showed, that sidedness represents a factor significantly associated with OS (HR 1.53; 95%CI 1.04–2.25; p = 0.029) alongside other well-known factors like lymph node status and extent of intra- and extrahepatic tumour involvement even when correcting for confounders such as age [17, 18, 21]. This association of RCRC and worse OS was marginally not statistically significant when excluding the two patients (HR 1.45; 95%CI 0.98–2.15; p = 0.061). However, when looking at the HR and CI in detail, the impact of all included variables in the model did no change in a clinically relevant magnitude whether these two cases were included or not.

To finally establish the oncological value of sidedness on survival after CRLM resection a meta-analysis with 10 studies including a total of 4312 patients was performed. The significant association of RCRC with worse OS was also confirmed in this meta-analysis. However, geographical sub-analysis, showed, that this association does not apply for Asian patients reported in two studies. For the whole cohort, interestingly, the resulting total HR of 1.55 is identical to the effect calculated in a meta-analysis of first-line chemotherapy trials presented by the Munich oncology group of Heinemann [35]. Similar to their analysis, we also did not find a relevant publication bias (Fig 4B and Fig 5B), although in our setting only retrospective cohort analysis were available. Regarding the impact of sidedness on recurrence after resection for CRLM, evidence from our own study as well as from existing literature is less clear. In the present cohort, although LCRC showed an increased median DFS of 12.1 vs. 9.1 months compared to RCRC, this was not statistically significant (p = 0.078). Furthermore, when correcting for other factors in multivariable analysis, although a tendency towards increased recurrence in RCRC was shown (HR 1.33; 95%CI 0.95–1.87), this was not statistically significant either (p = 0.099). Some other studies that included results in the context of sidedness and DFS after CRLM resection even suggested a beneficial effect for patients with LCRC [44, 51]. Accordingly, the present meta-analysis with considerable inhomogeneity did not confirm a clear statistically significant association with RCRC and worse DFS in the random effects model (HR 1.22; 95%CI 0.98–1.51). However, this result may in part be influenced by an Northern American study of Sasaki et al. acting as a statistical outlier outside the 95% CI in the funnel plot, thereby limiting the final conclusions regarding DFS [44]. The authors of this study proposed a possible explanation of the contradictory results in terms of OS and DFS: In their detailed analysis of recurrence patterns, patients with RCRC experienced more advanced extent of relapse despite a time to recurrence similar to LCRC patients. Moreover, intriguingly the geographical meta-analysis subgroup evaluation showed, that patients in the two European studies (Goto et al. and our data) were at the border of a statistically significant association of RCRC and worse DFS (HR 1.32; 95%CI 1.00–1.73; p = 0.05). Also, no Asian studies reported outcome on DFS. Therefore, potential racial or geographical DFS disparities might not be fully illustrated in the currently available, limited literature.

To evaluate the clinical applicability of sidedness as a factor for risk stratification in patients undergoing CRLM resection, we exemplarily created a clinical risk score derived from the 3 factors most significantly associated with OS in multivariable analysis and non-time-dependent ROC analysis (lymph node positivity, RCRC, extrahepatic involvement). Intriguingly, through time-dependent survival analysis (Kaplan-Meier and timeROC) we could show that this score was able to predict not only OS but also DFS in our two-institutional cohort as well as in an independent, international validation set (Fig 2A–2D). Although its’ discriminative ability is of moderate strength with a time-dependent AUC between 0.701 and 0.615 for OS within 12–60 months and 0.561 and 0.610 for DFS, these values are comparable to or better than those of other scores that have been extensively used in the past years. For example, the well-established “Fong-score” (MSKCC traditional risk score [17]) has recently been re-evaluated in a large cohort study from MD Anderson with an AUC of 0.57 (95%CI 0.48–0.65) for OS and 0.58 (95%CI 0.47–0.68) for DFS at five years postoperatively, which has also been confirmed through large multicentre validation cohort [21]. In comparison to the Fong-score, our risk score comprises only 3 instead of 5 factors. In principle it is also derivable from purely preoperatively available information, since lymph node involvement is nowadays often determined on preoperative imaging or–in case of metachronous metastases surgery already histologically confirmed by the time of liver resection. Furthermore, compared to mutational status analysis (RAS or BRAF) included in recently proposed clinical risk scores [20, 21], integrating sidedness gives an appealingly simple possibility to indirectly include information on tumour biology into preoperative stratification. However, the discriminative ability of these newer mutational clinical risk scores seems to exceed scores such as ours purely derived by dichotomous clinical variables. Furthermore, as a result of the limited number of patients with all three risk factors present (RCRC, nodal positivity and EHD), this highest-risk group is comparably small in size in both the test and validation set (<2% of cases). Furthermore, discrimination between two and three risk factors is limited. Accordingly, in case of further prospective external score validation in a large international multicentre-cohort, pooling patients with two and three risk factors into one high-risk group could be useful. In summary, the strength of sidedness in clinical risk prediction lies in fast and easy availability, for example during multidisciplinary tumour boards where an approximation of expected survival after resection for CRLM is often useful during individual case discussions. Similarly, our risk score is easy applicable in clinical practice and may also be used in stratification for trials on preoperative systemic treatment for CRLM. The value of the risk score is exemplarily shown by the major difference in median OS between all patients with three factors (21.7 months) compared to those with none of the implemented factors present (62.1 months), an almost threefold increase in survival.

The exact anatomical demarcation dividing right- versus left-sided tumours remains a matter of debate since molecular features change gradually along the colo-rectum [15, 57, 58]. As a matter of practicability, a dichotomous approach with division at the splenic flexure has been chosen in almost all previous reports on this topic. Furthermore, interpreting the rectum as an own biological entity has been proposed by some previous authors. Since several groups such as Dupré et al. and Wang et al. in their individual analysis of RCRC vs. LCRC did not find a difference in OS when rectal cancers were excluded, we decided to follow the same principal classifying RCRC vs. LCRC (including the rectum) in our cohort analysis [14, 15, 44]. However, it should be noted, that Dupré et al. found differences regarding the DFS if patients with rectal cancer were excluded from the analysis [15]. Furthermore, in our own cohort, left-sided colon and rectum tumours had similar OS, while right-sided colon tumours were markedly worse. On the other side, although also DFS was the worst in right-sided tumours, rectum tumours showed comparably poor DFS, while left-sided colon cancers were markedly better. Accounting for these details, we performed an additional meta-analysis with only those studies that excluded rectal cancer patients. This sub-analysis (provided as supplement) not only confirmed our previous findings with markedly inferior OS for RCRC patients (HR 1.62; 95%CI 1.28–2.04) but also resulted in a significant difference regarding DFS (1.39; 95%CI 1.10–1.76).

Most of the limitations of the current study originate from its’ retrospective nature. Firstly, mutational status analysis (RAS, BRAF) was only recently established as a routine examination in both institutions and therefore data was only incompletely available. Without any doubt, these markers are more and more becoming key factors in personalised oncosurgical treatment of mCRC patients [12, 19–21, 27, 34, 45]. However, in an era of excessive increase in cost for oncological diagnostics and therapies, we strongly believe, that simple affordable clinical risk factors such as lymph node status, extent of disease on imaging as well as primary tumour sidedness will continue to play a strong role especially in but not limited to less-developed health-care system settings. Details on the histological subtype of the primary tumour were also not readily obtainable for all cases, since a significant number of patients underwent primary resection outside our departments. This referral of advanced metastatic patients to specialized hepatobiliary centres reflects a typical pattern in many countries. Hence, the lack of histological information has been acknowledged in a number of comparable reports [14, 15, 44]. Future studies should incorporate this factor to further analyse the biological differences between RCRC and LCRC patients and survival after CRLM resection. Finally, evaluation of only resectable patients may generate a relevant bias in published studies. However, these studies as well as ours were primarily designed to examine survival after resection and the observed association of sidedness with survival was almost identical to meta-analysis of first line palliative chemotherapy trials.

## Conclusions

Patients with CRLM of right-sided primary colon cancer experience worse survival after hepatic resection than left-sided CRC patients. While the association with OS has been demonstrated in an own cohort with international validation and through meta-analysis, DFS is only significantly worse in RCRC compared to left-sided tumours when rectal cancer patients are excluded. The difference in survival is accounted for by the more aggressive biological behaviour of RCRC liver metastases with higher rates of mutations in oncogenes such as RAS and BRAF and probably other factors associated with the histological subtype of primary tumour and surgical outcomes of simultaneous colonic resection that have not yet been analysed prospectively in detail by the oncosurgical scientific community. Incorporating primary tumour sidedness into clinical risk stratification along other established variables is an easy and effective way to determine the mCRC patients’ postoperative prognosis in case of planned resection for CRLM.

## Supporting information

S1 Table Data table for all studies (n = 10) included in meta-analysis on sidedness and overall survival. (DOC)

S2 Table Data table for all studies (n = 6) included in meta-analysis on sidedness and disease-free survival. (DOC)

S3 Table Data table for all studies excluding rectal cancer patients (n = 5) included in meta-analysis on sidedness and overall survival. (DOC)

S4 Table Data table for all studies excluding rectal cancer patients (n = 5) included in meta-analysis on sidedness and disease-free survival. (DOC)

S5 Table PRISMA 2009 checklist of meta-analysis. (DOC)

S1 Fig Association of right-sided tumour location with overall survival (meta-analysis). Subanalysis according to geographical region of patient inclusion.
(TIF)

S2 Fig Association of right-sided tumour location with disease-free survival (meta-analysis). Subanalysis according to geographical region of patient inclusion.
(TIF)
