# Supplementary material for: Primary tumour location affects survival after resection of colorectal liver metastases: A two-institutional cohort study with international validation, systematic meta-analysis and a clinical risk score
Source: PLoS One. 2019 May 31;14(5):e0217411. doi: 10.1371/journal.pone.0217411 (PMC6544347; doi:10.1371/journal.pone.0217411)
Supplement: Supplementary file 2 [file pone.0217411.s001.doc]

Meta-analysis: generic inverse variance method




# Meta-analysis: generic inverse variance method

|  |  |
| --- | --- |
| Variable for studies | Study |
| Variable for estimate: | ln\_oshr |
| Variable for Standard Error: | SE\_ln\_oshr |

 

|  |  |  |  |  |  |  |  |  |
| --- | --- | --- | --- | --- | --- | --- | --- | --- |
| Study | Estimate (Log) | Standard Error | Estimate | 95% CI | z | P | Weight (%) | |
| Fixed | Random |
| Creasy et al. | 0,199 | 0,0881 | 1,220 | 1,026 to 1,450 |  |  | 30,48 | 15,41 |
| Dupré et al. | 0,642 | 0,223 | 1,900 | 1,228 to 2,940 |  |  | 4,77 | 7,50 |
| Gasser et al. | 0,425 | 0,197 | 1,530 | 1,040 to 2,250 |  |  | 6,11 | 8,66 |
| Lee-Ying et al | 0,336 | 0,156 | 1,400 | 1,032 to 1,900 |  |  | 9,75 | 10,88 |
| Marques et al. with KRAS (=mut) | 0,742 | 0,701 | 2,100 | 0,531 to 8,300 |  |  | 0,48 | 1,17 |
| Marques et al. without KRAS (=wt) | 0,0953 | 0,439 | 1,100 | 0,465 to 2,600 |  |  | 1,23 | 2,73 |
| Sasaki et al. | 0,344 | 0,168 | 1,410 | 1,014 to 1,960 |  |  | 8,38 | 10,17 |
| Wang et al. | 0,0770 | 0,178 | 1,080 | 0,762 to 1,530 |  |  | 7,49 | 9,63 |
| Yamashita et al. (preoperative CTx) | 0,713 | 0,122 | 2,040 | 1,607 to 2,590 |  |  | 15,96 | 13,08 |
| Yamashita et al. (no properative Ctx) | 0,642 | 0,192 | 1,900 | 1,303 to 2,770 |  |  | 6,40 | 8,88 |
| Goto et al. (PSM) | 0,582 | 0,170 | 1,790 | 1,282 to 2,500 |  |  | 8,15 | 10,03 |
| Imai et al. | 1,235 | 0,546 | 3,440 | 1,180 to 10,030 |  |  | 0,79 | 1,85 |
| Total (fixed effects) | 0,401 | 0,0487 | 1,494 | 1,358 to 1,643 | 8,251 | <0,001 | 100,00 | 100,00 |
| Total (random effects) | 0,437 | 0,0782 | 1,549 | 1,329 to 1,805 | 5,591 | <0,001 | 100,00 | 100,00 |

## Test for heterogeneity

|  |  |
| --- | --- |
| Q | 22,3770 |
| DF | 11 |
| Significance level | P = 0,0216 |
| I^2^ (inconsistency) | 50,84% |
| 95% CI for I^2^ | 4,84 to 74,61 |
