# Supplementary material for: Primary tumour location affects survival after resection of colorectal liver metastases: A two-institutional cohort study with international validation, systematic meta-analysis and a clinical risk score
Source: PLoS One. 2019 May 31;14(5):e0217411. doi: 10.1371/journal.pone.0217411 (PMC6544347; doi:10.1371/journal.pone.0217411)
Supplement: Supplementary file 3 [file pone.0217411.s002.doc]

Meta-analysis: generic inverse variance method




# Meta-analysis: generic inverse variance method

|  |  |
| --- | --- |
| Variable for studies | Study |
| Variable for estimate: | ln\_dfshr |
| Variable for Standard Error: | SE\_ln\_dfshr |

 

|  |  |  |  |  |  |  |  |  |
| --- | --- | --- | --- | --- | --- | --- | --- | --- |
| Study | Estimate (Log) | Standard Error | Estimate | 95% CI | z | P | Weight (%) | |
| Fixed | Random |
| Creasy et al. | 0,131 | 0,0863 | 1,140 | 0,963 to 1,350 |  |  | 34,72 | 17,75 |
| Gasser et al. | 0,285 | 0,174 | 1,330 | 0,946 to 1,870 |  |  | 8,55 | 13,33 |
| Marques et al. with KRAS (=mut) | 0,0953 | 0,419 | 1,100 | 0,484 to 2,500 |  |  | 1,47 | 5,15 |
| Marques et al. without KRAS (=wt) | 0,000 | 0,300 | 1,000 | 0,556 to 1,800 |  |  | 2,87 | 8,06 |
| Sasaki et al. | -0,288 | 0,147 | 0,750 | 0,562 to 1,000 |  |  | 11,99 | 14,73 |
| Yamashita et al. (preoperative CTx) | 0,536 | 0,0975 | 1,710 | 1,413 to 2,070 |  |  | 27,19 | 17,23 |
| Yamashita et al. (no properative Ctx) | 0,392 | 0,174 | 1,480 | 1,053 to 2,080 |  |  | 8,57 | 13,34 |
| Goto et al. (PSM) | 0,255 | 0,236 | 1,290 | 0,812 to 2,050 |  |  | 4,63 | 10,41 |
| Total (fixed effects) | 0,228 | 0,0508 | 1,256 | 1,137 to 1,388 | 4,486 | <0,001 | 100,00 | 100,00 |
| Total (random effects) | 0,195 | 0,110 | 1,215 | 0,979 to 1,509 | 1,767 | 0,077 | 100,00 | 100,00 |

## Test for heterogeneity

|  |  |
| --- | --- |
| Q | 25,3151 |
| DF | 7 |
| Significance level | P = 0,0007 |
| I^2^ (inconsistency) | 72,35% |
| 95% CI for I^2^ | 43,31 to 86,51 |
