# Supplementary material for: Primary tumour location affects survival after resection of colorectal liver metastases: A two-institutional cohort study with international validation, systematic meta-analysis and a clinical risk score
Source: PLoS One. 2019 May 31;14(5):e0217411. doi: 10.1371/journal.pone.0217411 (PMC6544347; doi:10.1371/journal.pone.0217411)
Supplement: Supplementary file 4 [file pone.0217411.s003.doc]

Meta-analysis: generic inverse variance method




# Meta-analysis: generic inverse variance method

|  |  |
| --- | --- |
| Variable for studies | Study |
| Variable for estimate: | ln\_oshr |
| Variable for Standard Error: | SE\_ln\_oshr |

 

|  |  |  |  |  |  |  |  |  |
| --- | --- | --- | --- | --- | --- | --- | --- | --- |
| Study | Estimate (Log) | Standard Error | Estimate | 95% CI | z | P | Weight (%) | |
| Fixed | Random |
| Creasy et al. | 0,199 | 0,0881 | 1,220 | 1,026 to 1,450 |  |  | 43,09 | 24,68 |
| Lee-Ying et al | 0,336 | 0,156 | 1,400 | 1,032 to 1,900 |  |  | 13,78 | 19,12 |
| Yamashita et al. (preoperative CTx) | 0,713 | 0,122 | 2,040 | 1,607 to 2,590 |  |  | 22,56 | 21,95 |
| Yamashita et al. (no properative Ctx) | 0,642 | 0,192 | 1,900 | 1,303 to 2,770 |  |  | 9,04 | 16,30 |
| Goto et al. (PSM) | 0,582 | 0,170 | 1,790 | 1,282 to 2,500 |  |  | 11,52 | 17,95 |
| Total (fixed effects) | 0,418 | 0,0578 | 1,519 | 1,356 to 1,701 | 7,226 | <0,001 | 100,00 | 100,00 |
| Total (random effects) | 0,479 | 0,118 | 1,615 | 1,280 to 2,036 | 4,044 | <0,001 | 100,00 | 100,00 |

## Test for heterogeneity

|  |  |
| --- | --- |
| Q | 14,6066 |
| DF | 4 |
| Significance level | P = 0,0056 |
| I^2^ (inconsistency) | 72,62% |
| 95% CI for I^2^ | 31,28 to 89,09 |
