# Supplementary material for: Primary tumour location affects survival after resection of colorectal liver metastases: A two-institutional cohort study with international validation, systematic meta-analysis and a clinical risk score
Source: PLoS One. 2019 May 31;14(5):e0217411. doi: 10.1371/journal.pone.0217411 (PMC6544347; doi:10.1371/journal.pone.0217411)
Supplement: Supplementary file 5 [file pone.0217411.s004.doc]

Meta-analysis: generic inverse variance method




# Meta-analysis: generic inverse variance method

|  |  |
| --- | --- |
| Variable for studies | Study |
| Variable for estimate: | ln\_dfshr |
| Variable for Standard Error: | SE\_ln\_dfshr |

 

|  |  |  |  |  |  |  |  |  |
| --- | --- | --- | --- | --- | --- | --- | --- | --- |
| Study | Estimate (Log) | Standard Error | Estimate | 95% CI | z | P | Weight (%) | |
| Fixed | Random |
| Creasy et al. | 0,131 | 0,0863 | 1,140 | 0,963 to 1,350 |  |  | 46,23 | 32,23 |
| Yamashita et al. (preoperative CTx) | 0,536 | 0,0975 | 1,710 | 1,413 to 2,070 |  |  | 36,20 | 30,82 |
| Yamashita et al. (no properative Ctx) | 0,392 | 0,174 | 1,480 | 1,053 to 2,080 |  |  | 11,41 | 21,42 |
| Goto et al. (PSM) | 0,255 | 0,236 | 1,290 | 0,812 to 2,050 |  |  | 6,16 | 15,53 |
| Total (fixed effects) | 0,315 | 0,0587 | 1,371 | 1,222 to 1,538 | 5,374 | <0,001 | 100,00 | 100,00 |
| Total (random effects) | 0,331 | 0,120 | 1,392 | 1,100 to 1,763 | 2,749 | 0,006 | 100,00 | 100,00 |

## Test for heterogeneity

|  |  |
| --- | --- |
| Q | 9,9735 |
| DF | 3 |
| Significance level | P = 0,0188 |
| I^2^ (inconsistency) | 69,92% |
| 95% CI for I^2^ | 13,54 to 89,53 |
